# The metrics of reading speed: understanding developmental dyslexia

**DOI:** 10.1038/s41598-024-52330-x

**Published:** 2024-02-19

**Authors:** Sara Conforti, Chiara Valeria Marinelli, Pierluigi Zoccolotti, Marialuisa Martelli

**Affiliations:** 1 Department of Psychology, Sapienza University of Rome, Rome, Italy; 2 Cognitive and Affective Neuroscience Lab, Department of Humanities, University of Foggia, Foggia, Italy; 3 Tuscany Rehabilitation Clinic, Montevarchi, Italy

**Keywords:** Psychology, Human behaviour

## Abstract

We compared reading words and pseudo-words presented in single displays (as typical of psycholinguistic research) with stimuli presented in multiple displays (as typical of real-life conditions and clinical testing) under controlled conditions. Italian sixth-grade children with and without a reading deficit showed an advantage in reading times for multiple over single displays. This finding was partly ascribed to the capacity to overlap the non-decisional component of the response, an effect present in control readers as well as children with dyslexia. Furthermore, there were several indications in the data that the requirement to read sequentially taxes performance by augmenting the relative impact of the experimental manipulations used. This effect was present in both groups of children, but proportionally stronger in children with dyslexia. The study contributes to filling the gap between single and multiple displays, a condition more like real-life situations.

## Introduction

Children with developmental dyslexia are unimpaired in reading single letters^1^ even after controlling for differences in difficulty between reading single letters and words^2^. Instead, they selectively fail in processing strings of letters (whether words or pseudowords), indicating that the nuclear deficit in developmental dyslexia is first at the level of orthographic string decoding^3^. Indeed, major models of reading (such as the dual-route cascaded model, DRC^4^; the CDP + model^5^; and the triangle model^6^) focus on explaining the reading behaviour at the single-word level. However, in everyday school activities, children must manage meaningful texts. In standard text reading, the need to read words in a sequence and integrate this processing with other sub-components of reading (such as holding in memory the word to be pronounced) may represent an additional burden, selectively aggravating the reading of children with dyslexia. The general goal of the present study was to model the reading performance of children with dyslexia and skilled readers in the case of multiple displays, as typical of functional reading.

*Rapid automatized naming (RAN)*. One reason to imagine that there is something beyond the “orthographic processing” level is the strong correlation between reading fluency and performance in Rapid Automatized Naming (RAN) tasks^7^. RAN correlates with reading more when stimuli (figures, colours, or digits) appear simultaneously on a single sheet, and the subject is requested to name them serially rather than in discrete form, i.e., with individual items singly presented (e.g., ^8–11^). This format specificity emerges in the course of development; thus, in first grade, the standard serial RAN correlates with reading irrespective of the format of the measure of reading^9^. Later, discrete RAN correlates more with single than multiple-word reading while serial RAN correlates more with serial than discrete reading^9,13^. The relationship between RAN and reading can reveal important information on the development of reading fluency, attracting reading research on the distinction between serial and discrete processing (e.g., ^9–17^).

*Comparing reading from single vs multiple displays*. The comparison between single, discrete word displays (typical of experimental settings) and multiple displays (as it occurs in the reading of meaningful texts and clinical settings) may help in our understanding of reading mechanisms in a functional context, and this is the research perspective pursued in the present study. However, up to date, this approach has been used in only a few studies^16,17^, while most previous research adopted a correlational approach ^9–15^.

Directly comparing single and multiple reading displays presents several methodological problems *in primis* due to variations in the general difficulty of the two tasks. Furthermore, different measures are typically used in the two cases at hand.

The frequently used measure to examine performance in reading single words is vocal reaction time (RT). RTs refer to the time interval between the target stimulus onset and the beginning of the response. In the case of lists of words or texts, the frequently used measure is reading time, which also includes the time to utter the stimulus (pronunciation time). Therefore, if one wants to compare reading performance across single and multiple displays, one possibility is to use reading times also in the case of single word displays, i.e., to measure not only RTs but also pronunciation times. In general, reaction times are more sensitive to the decoding component of the processing than the pronunciation times. Thus, they are the elective measure when examining the effect of psycholinguistic parameters in word recognition. An additional reason is that RTs can be detected using standard automatic procedures, while measuring pronunciation times (or reading times to individual targets) requires time-consuming single-trial analyses. Finally, there is much knowledge on the rules (or laws) that govern RTs (e.g., ^18^), and several models have been proposed to account for individual performance under timed conditions (e.g., ^19,20^).

The most frequently used measure in the case of multiple displays is the overall time to read or name the list of stimuli. Thus, decoding and pronunciation are combined within a single measure and cannot be easily disentangled^17^. Depending on the research aim, the overall time to read a list of words or a text is then divided by the number of relevant items (e.g., words or syllables) to obtain a measure of time per item (i.e., s/word or s/syllable). In Italian children, the language object of the present study, these speed measures highlight a reading delay of a factor of about 2 in children with dyslexia (for a meta-analysis^21^). This measure describes the deficit severity across studies and types of reading tests used because it relates the amount of time spent reading different texts/lists to the same unit of measurement (e.g., word or syllable). However, reading time includes both decoding and pronunciation time, and as such, it does not allow for distinguishing the source of the deficit.

Furthermore, investigations into single-word reading typically measure the effect of psycholinguistic variables (such as word frequency, lexicality, etc.) and examine group differences as a function of such variables. However, various models aim to account for individual performance in speeded tasks. Below, we illustrate how reference to three such models (i.e., difference engine model, DEM^19^; rate and amount model, RAM^20^; and state trace analysis^22^) may help frame the study of reading in single versus multiple display conditions, which, to anticipate, represents the general goal of the present research.

A common feature of these approaches is that they focus on detecting global (i.e., not task-specific) components in the experimental data. Thus, tests based on these models may allow a comprehensive evaluation of the influence of global components in shaping the reading times as a function of group and type of display (for a similar approach on aging, see Verhaeghen & Cerella^23^). Below, we illustrate the general characteristics of these three approaches and describe how they can be informative in comparing single and multiple reading displays.

*Relationship between condition means and variability as a basic “rule” of individual variability*. The “*difference engine model*” (DEM)^19^ starts from the observation that in RT studies, variability across conditions typically grows as a function of condition difficulty over and above the influence of specific experimental conditions. This relationship would represent a basic rule accounting for individual variability in speeded tasks^19^ (see also^18^). Based on this plot, Myerson et al.^19^ identify two separate and independent portions of the response, named “*compartments*”. The cognitive compartment identifies the part of the response due to decisional time, differentiating two groups of individuals with a global difference in processing efficiency across different tasks (e.g., young adults versus elderly^24^). The “sensory-motor” compartment identifies the portion of the time required for peripheral analysis and programming/beginning of the motor response. The cognitive compartment is indicated by near-linearity in the plot, contrasting condition means with their corresponding standard deviations. The “sensory-motor” compartment is measured as the intercept on the x-axis of the means-SD plot (for more information on DEM, please refer to Supplementary Materials).

In a study based on DEM, Martelli et al.^24^ separately measured the components that contribute to reading times in the case of single-word reading, that is, decoding and pronunciation, and evaluated whether (and to what extent) pronunciation times contributed to group differences. Manipulation of target length and lexicality allowed varying task difficulty to assess the cognitive compartment in reading. Results from this study are instrumental in understanding the key differences between RT and reading time measures in single-word reading. This represents a heuristic basis for developing predictions on the influence of multiple displays in modulating the reading times as a function of factors such as word length and frequency, which is one of the aims of the present study.

**Figure 1:**
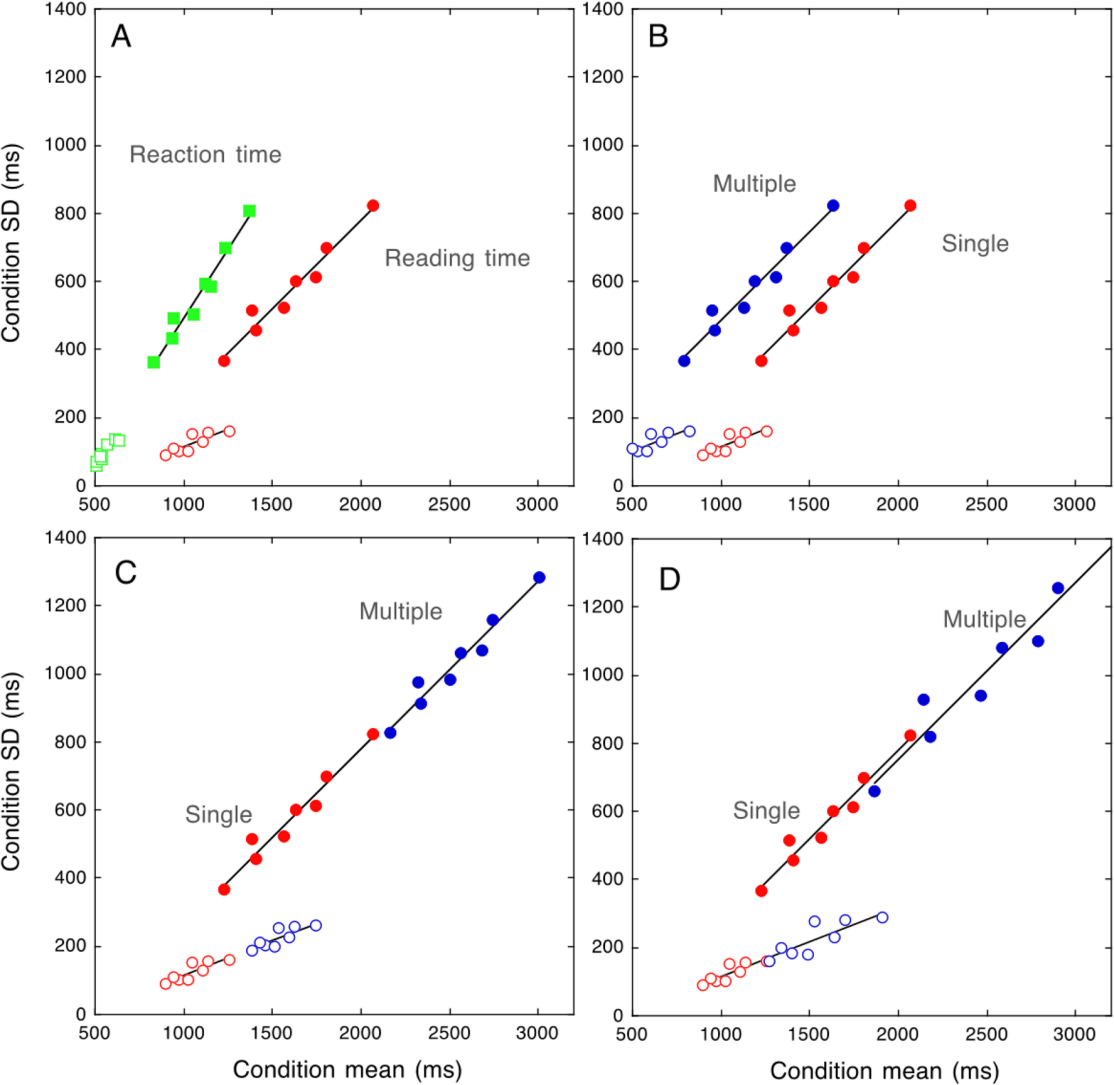
Plot (**A**) shows RTs and reading times of children with dyslexia (filled symbols) and control children (open symbols) in reading single words varying for length and frequency (data derived from Martelli et al.^24^). Note that (**A**) the slope between means and SDs is larger for vocal RTs than reading times. Pronunciation time (although longer for longer words) adds a constant factor independent of group diluting the effect of experimental conditions (thus producing shallower slopes than those obtained from vRTs measures); (**B**), the overall level of inter-individual variability does not change appreciably (as pronunciation times add only a minimal proportion of variability); (**C**) even though pronunciation times are similar in the two groups, they weigh proportionally more in the case of typically developing children and less in the case of children with dyslexia (whose reading times are proportionally more influenced by their longer RTs). Thus, in the case of reading times, two separate regression lines are necessary to account for the relationship between means and SDs in typically developing children and in children with dyslexia. Plots 1 (**B**) to 1 (**D**) show hypothetical changes in reading times between single and multiple displays. Data for single displays (only reading times) as in Plot 1 (**A**). In Plot 1 (**B**), data for multiple displays are shifted toward the left, i.e., with a reduction in the intercept but no change in slope. In Plot 1 (**C**), SDs and means are increased in the multiple display conditions by a fixed amount. In plot 1 (**D**), SDs and means increase proportionally to condition difficulty with a corresponding increase in the spread of the distribution.

The relationship between SDs and condition means from the data of Martelli et al.^24^ is re-plotted in Fig. 1A. In keeping with the DEM’s predictions for RTs, the relationship grows linearly for both typically developing children and children with dyslexia; a single regression line well explains this relationship in both groups. Figure 1A also shows the total reading times of the two groups of children, i.e., a measure that includes the reading pronunciation times (not separately shown in the figure). To understand the changes in the relationship between means and SDs and between RTs and reading times, we need to consider some peculiar characteristics of pronunciation times. They: (a) yield extremely small individual differences: indeed, the reported error terms were 15 times (or more) smaller than those in vRTs or reading times; (b) are very similar between the two groups of children; and (c) vary as a function of length and frequency (e.g., they are slower for longer words) with no effect on SDs^24^. In short, they are nearly constant between groups while they vary across experimental conditions. These characteristics are relevant in accounting for the pattern of reading times^24^.

The reading times in Fig. 1A (next called “single”) represent the basis for developing predictions for reading stimuli presented in multiple displays. In doing so, we only loosely refer to the general principles of the DEM^19^ as this model is not aimed to predict either reading times to multiple or single displays (rather than RTs). Figures 1B–D illustrate some of these predictions. We know that, in the case of multiple displays, reading times tend to be faster, at least in the case of typically developing children e.g.,^17^. One possibility to account for this is that reading times for multiple items are merely shifted to the left (i.e., with a change in the intercept on the x-axis), as illustrated in Fig. 1B. A way to interpret this pattern is that the integration among the various sub-components of reading (such as exploring the text, recognizing the words, and keeping the response in short-term memory) is made possible primarily by the overlap of the sensory-motor compartment of the response. However, there is reason to believe that the shift to the left (envisaged in Fig. 1B) would not suffice to account for all the variations observed in the case of reading in multiple displays. Zoccolotti et al.^17^ noted that reading times of multiple displays were also associated with larger SDs, an effect particularly clear in the case of children with dyslexia. Furthermore, Protopapas et al.^15^ noted that word fluency “*requires a complex skill of endogenous sequential processing over and above individual word recognition*”. This is defined as cascaded processing and refers to the idea that different processing stages (both parallel and sequential) may partially overlap temporally, influencing the elaboration of items in a sequence^12^. Thus, one may hypothesize that reading from multiple displays might amplify the effect of the experimental variables, increasing both means and SDs. One may expect two different outcomes illustrated in Fig. 1C and 1D, respectively. Plot 1C illustrates a possible additive effect of multiple over single display conditions. SDs are increased in the multiple-display conditions by a fixed amount; condition means are also increased by a fixed amount netted from the sensory-motor component, producing a shift of the data on the regression line with no change in the relative effect of the variables manipulated. Plot 1D illustrates a possible multiplicative effect of multiple over single display conditions. The increases in SDs and the means are proportional to task difficulty, yielding a change in the spread of the distribution. One may also anticipate that the changes in the performance pattern with multiple stimuli may involve a mix of the changes shown in plots 1B, 1C, and 1D.

*Testing the over-additivity effect in group differences using the Brinley plot analysis (RAM)*. A problem in comparing target groups of individuals is to reliably establish the size of their performance differences as a function of a variety of experimental conditions. One model aimed to deal with this problem in timed tasks is the *rate and amount model* (RAM^20^). RAM proposes that, at individual and group levels, RTs largely depend on two factors: the general cognitive speed of the individual (rate) and the difficulty of a given condition (amount). These two factors interact multiplicatively to determine individual performance. As an effect of this, group differences tend to be larger in more difficult conditions, i.e., they tend to show an over-additivity effect (for more information on RAM, please refer to Supplementary Materials).

Based on Brinley plot analysis, children with dyslexia show a marked, multiplicative (or over-additive) RT delay across stimulus materials and conditions (also referred to as “global factor”) in dealing with orthographic visual materials (e.g.,^3,24–26^). Children with dyslexia are selectively impaired when they must operate on a string of graphemes presented visually, although not in the case of single letters or bigrams.^3,25^ This slowness cascades on all subsequent processing and amplifies group differences as a function of general condition difficulty.

**Figure 2:**
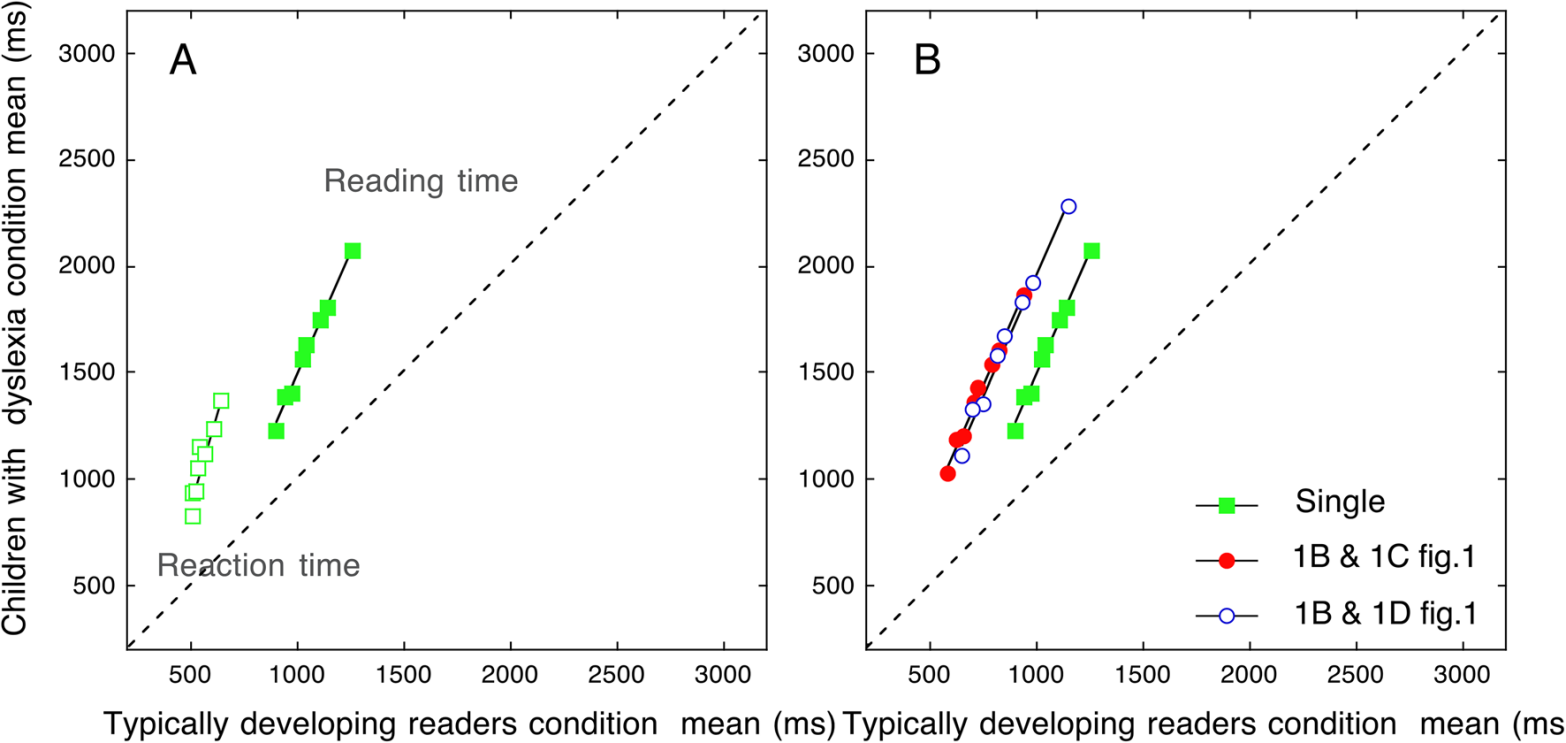
Brinley plots: condition means for children with dyslexia are shown as a function of the means obtained by typically developing readers. Slopes greater than 1 are indicative of an over-additive effect. Plot 2A shows the Brinley plots for reading (filled squares) and reaction time (open squares) measures in single displays (data derived from Martelli et al.^24^). A single regression line accounts well for the group differences in the case of vocal RTs (with a slope of 3.44; R2 = 0.88). A clear relationship is also present in the case of total reading times (R2 = 0.99) even though the slope is appreciably smaller (b = 2.3). The reduction in slope passing from RTs to total reading times is due to the diluting effect of including pronunciation times in this latter measure. Plot 2B shows predictions of reduced sensory-motor time and an additive (filled red) or multiplicative (open blue) increase in means and SDs for the multiple display conditions.

Data from Martelli et al.^24^ shown in Fig. 1A are represented in Fig. 2A as a Brinley plot. Again, these data represent a basis for studying reading times in the case of multiple displays. One may expect the multiple display to produce a shift along the x-axis due to compression of the sensory-motor component and an additive (solid red) or multiplicative (empty blue) effect on means and SDs relative to the single display condition (green squares, Fig. 2B).

**Figure 3:**
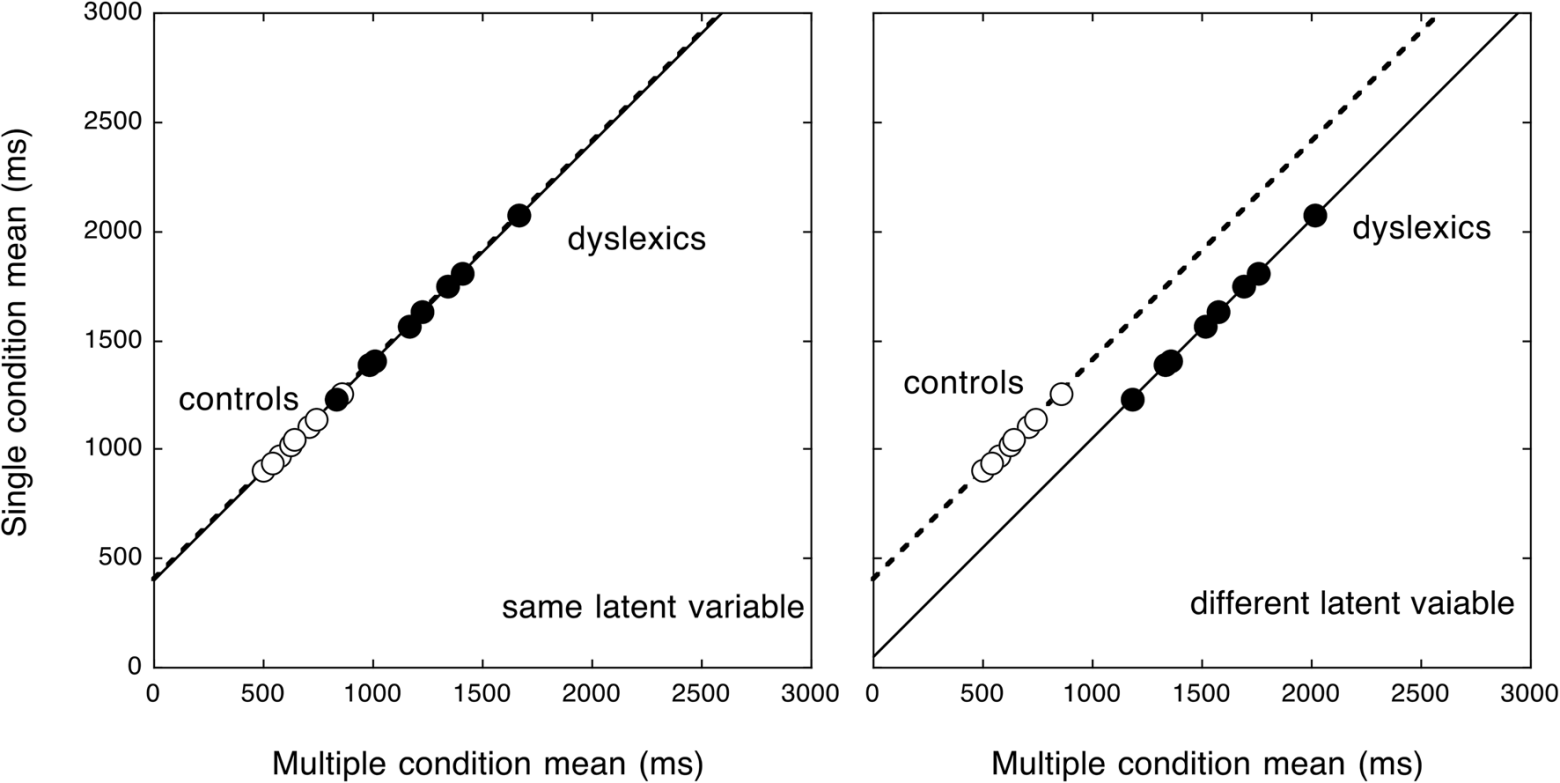
The state trace predictions (reading time data in the single display condition are redrawn from Martelli et al.^24^). If the same component is involved in both conditions, fast and slow readers should produce performance times captured by the same regression line (left panel). In the case of different cognitive components involved in the task, one would predict data to be captured by two regression lines (right panel).

*Relationship between single and multiple displays as a function of the reading deficit: State trace analysis*. Over-additivity highlights that standard statistical analyses (such as ANOVAs) are not well suited to compare two critical conditions across fast and slow populations, yielding different overall levels of performance and inter-individual variability^20,27^. One effective means to deal with this general problem is State trace analysis^22,28^, a relatively simple graphic way to define the space for a given process. In the State trace plot, one dependent variable is plotted as a function of another related variable. This analysis aims to determine whether the relationship between a set of causal and indicator variables (including a grouping variable^29^) can be ascribed to a single, latent variable or whether more variables should be invoked^28,29^ (for more information on State trace analysis, please refer to Supplementary Materials). We may expect a single latent component to explain the reading behaviour in the two display conditions (Fig. 3 left). Alternatively, Fig. 3 right depicts the prediction for diverse components involved in the two tasks between fast and slow readers.

### Goals of the present study

The present study aimed to test whether children with dyslexia would show any selective disadvantage in the multiple over single-display conditions. Significant group x condition interactions may indicate over-additivity effects. To reduce false positives in identifying group differences, we relied on three methods that allow controlling for global influences on the data. Previous research^16,17^ has already shown an advantage of multiple versus single displays, particularly in control children. Referring to models of global processing was expected to elucidate the internal structure of this advantage.

We used three procedures to examine the differences between typically developing readers and dyslexic children from a global (i.e., a non-task-specific) perspective. First, following the DEM^19^, we examined the relationship between the performance in experimental conditions and their corresponding SDs, referred to as the basic rule to account for individual variability in RT tasks. Second, following the RAM^20^, we used the *Brinley Plot* analysis to establish the size of the group differences across various experimental conditions, taking into account over-additive effects, i.e., the tendency to obtain larger group differences in the case of more difficult conditions over and above the effect of specific experimental manipulations. Altogether, these tests would allow for a comprehensive evaluation of the influence exerted by global components in shaping the reading times as a function of group and type of display. Finally, we examined the State trace between multiple and single display conditions^22^. This analysis aims to describe the relationship between these two tasks and to establish whether the performance of children with dyslexia and control readers can be ascribed to a single latent variable or if different variables need to be invoked.

## Results

### Descriptive analyses

**Figure 4:**
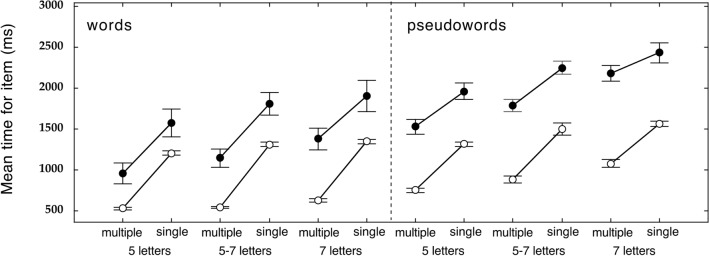
Mean raw total reading times (time per item in ms) and standard errors for typically developing readers (empty symbols) and children with dyslexia (filled symbols). Data are presented as a function of word length.

Figure 4 presents means and ESs for all experimental conditions (means and SDs are also reported in Supplementary Table S1). An inspection of the graphs (and Table) indicates that reading times:

- are always faster for words than pseudowords;
- vary as a function of stimulus length with shorter times for 5-letter targets, than slower for mixed 5–7-letter targets, and even slower for 7-letter targets;
- are consistently faster for stimuli presented in multiple than in single displays.

Some differences in the size of the advantage for multiple displays as a function of stimulus length and lexicality are apparent but difficult to appreciate as they are imbricated with differences in the general level of performance. However, they will emerge more clearly in analyses controlling for global components in the data.

### Detection of global components

**Figure 5:**
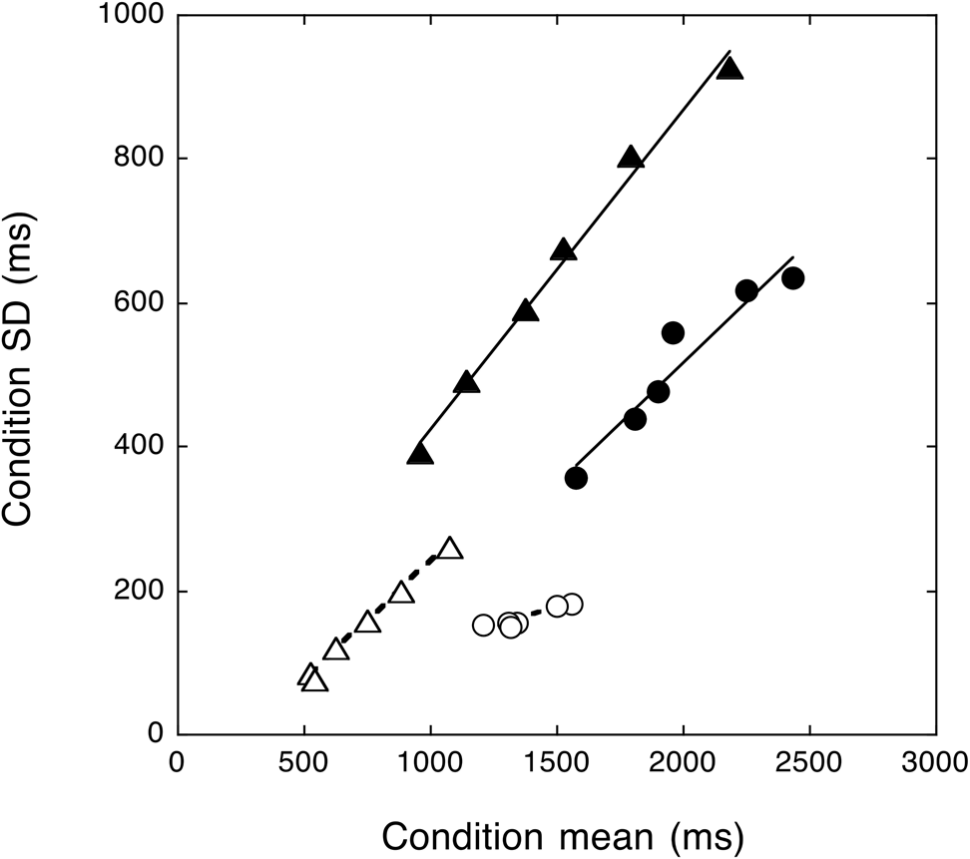
SDs of children with dyslexia (filled symbols) and typically developing readers (open symbols) are plotted as a function of multiple (triangles) and single (circles) conditions means in total reading times. Four regression lines, one for each group (controls and dyslexic children) and condition fit best the data. For controls in multiple display conditions, one regression line fits data (open triangles) with a slope of .33 (R2 = .99); for single display conditions the regression line fits data (open circles) with a slope of .10 (R2 = .88). For dyslexic children, in multiple display conditions, the regression line fits data (black triangles) with a slope of .44 (R2 = .99); for single display conditions, the regression line fits all data (black circles) with a slope of .34 (R2 = .93).

*Relationship between conditions means and interindividual variability (DEM)*. In Fig. 5, the SDs of children with dyslexia and controls are plotted as a function of the corresponding condition means of total reading times.

Inspection of the figure indicates several main findings:

- The present data on single display conditions closely follow the pattern originally reported in Martelli et al.^24^ for reading times to single word and pseudoword reading (see Fig. 1A, red dots).
- To account for the present data, four regression lines, one for each group (control and dyslexic children) and condition (single and multiple displays), are necessary. Fits of the regression lines are from good to very good for both groups of children and for both types of display conditions (range R^2^ = 0.88-0.99; for values, refer to the legend of Fig. 5).
- As predicted, individual variability generally grows as a function of condition difficulty, i.e., SDs are larger for slower conditions (a systematic violation of the homogeneity assumption required for parametric analyses). This tendency is attenuated in the case of the single display conditions for controls.
- Data for multiple display conditions are shifted to the left compared to single display conditions, indicating faster reading times in the former conditions for both groups of children.
- In the case of children with dyslexia, this is associated with a marked reduction in intercept (which passed from 468 ms in the case of single display conditions to 41 ms in the case of multiple display conditions). For controls, this is associated with a change in the slope of the regression that was steeper (b = 0.33) for the multiple display conditions than for the single display conditions (b = 0.10).

It is possible to describe the changes which occur in multiple displays using the data for the single display condition as a “probe” and the DEM as a general interpretative framework. In both groups, reading times are faster for multiple display conditions (i.e., they are shifted to the left). However, this is not associated with smaller SDs, as it would occur if a single global factor would explain all data. Rather, two features are apparent:

- The spread between conditions tends to be greater for multiple than single display conditions both in terms of means and SDs;
- SDs tend to be greater for multiple conditions, particularly for the most difficult experimental conditions; this trend is present in both groups but proportionally greater in children with dyslexia.

Overall, in dealing with multiple stimuli, controls, as well as children with dyslexia, can speed up their reading by overlapping non-decisional components of their responses. At the same time, the requirement to read from multiple displays poses a challenge over the decisional part of the response, amplifying the modulating effect of variables, such as lexicality and length. These two general tendencies exert opposite influences, making it difficult to detect these effects in the raw data (as shown in Fig. 4).

**Figure 6:**
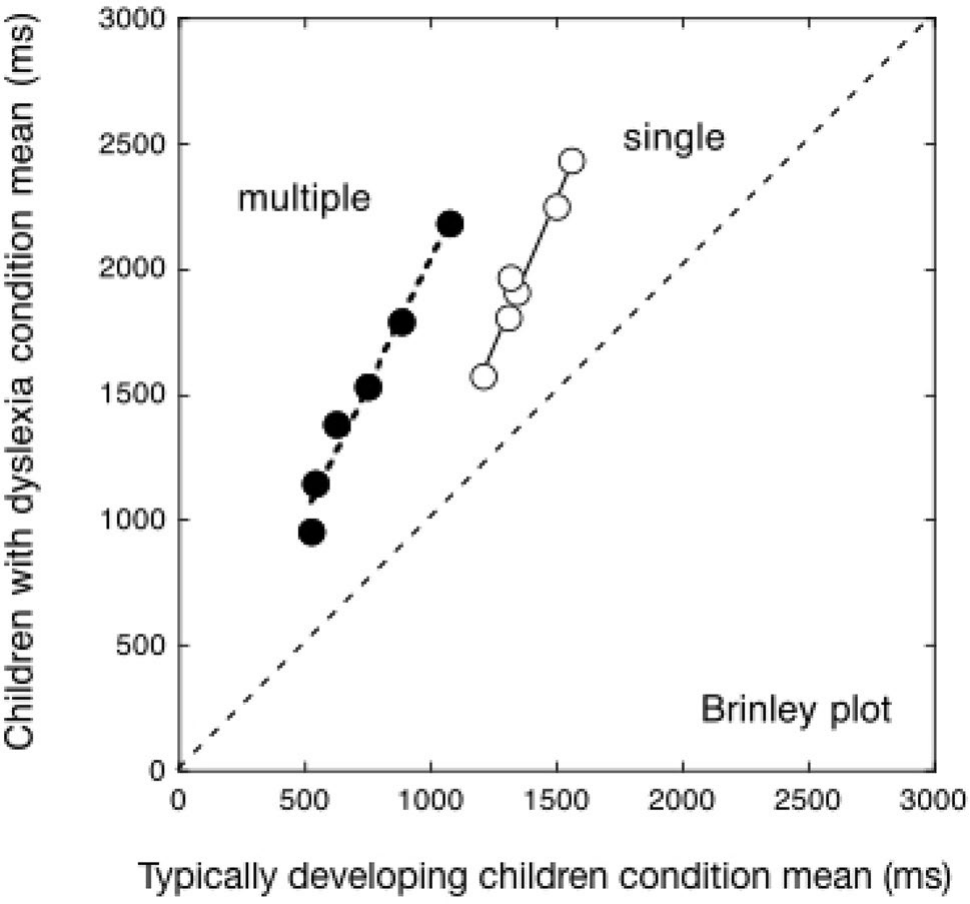
Brinley plot analysis: the condition means of the children with dyslexia are plotted against the respective condition means of the control children separately for the single display conditions (white circles) and the multiple display conditions (black circles). Two different regression lines, one for the multiple display condition (b = 2.04; R^2^ = 0.97) and one for the single display condition (b = 2.31; R^2^ = 0.97) fit well the experimental data. The diagonal line in the plot represents the equality line indicating no difference between the two groups.

*Brinley plot analysis*. Figure 6 presents experimental data as a Brinley plot.

A separate relationship is apparent for multiple and single display conditions. Group differences are accounted for by one regression line for the multiple-display conditions (with a beta equal to 2.04; R^2^ = 0.97) and one for the single-display conditions (with a beta of 2.31; R^2^ = 0.97). This pattern indicates an over-additivity effect; that is, children with dyslexia are more impaired than control readers in more difficult conditions over and above the influence of the specific experimental manipulations.

Note that the data points for multiple displays are shifted to the left, indicating two different intercepts on the y-axis. For single conditions, the intercept on the y-axis is negative (− 1188 ms) and crosses the diagonal line at a positive value of 913 ms; by contrast, for multiple conditions, the intercept on the y-axis approaches zero (− 4 ms) and crosses the equality line at 4 ms.

Two main findings emerge in the comparison between single and multiple display conditions. First, the slopes of the linear regressions for the single and multiple conditions are similar. Second, a clear difference between single and multiple display conditions emerges in the spread of the condition means; i.e., there are larger differences among conditions in the case of the multiple-display conditions than in the case of the single display conditions. Thus, the multiple-display conditions boost the effect of the experimental manipulations, e.g., they amplify the difficulty of children with dyslexia in reading long words.

**Figure 7:**
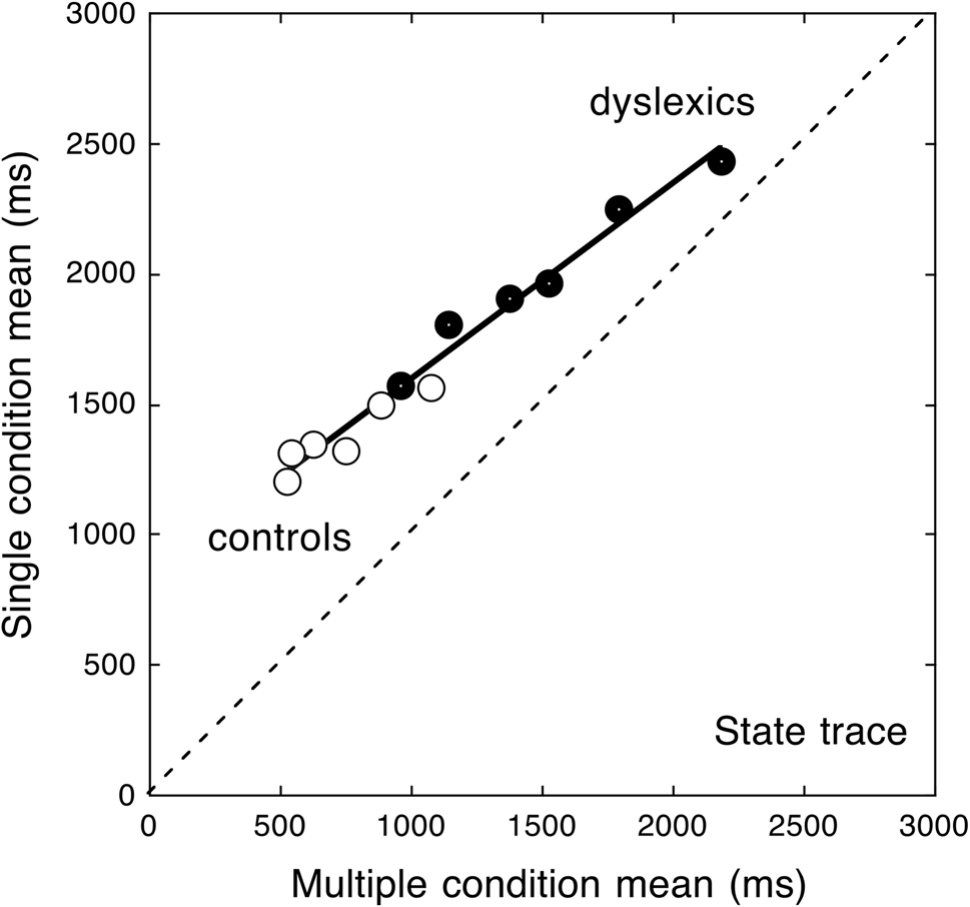
State trace analysis: the means of the multiple display condition are plotted against the means of the single display conditions for the children with dyslexia (filled circles) and for the control children (open circles). A single regression line explains well the data (a = 846 ms; b = 0.75; R2 = 0.98). The diagonal line in the plot represents the equality line indicating no difference between the two conditions.

*State trace analysis*. Figure 7 presents experimental data as a state trace plot. Inspection of the data indicates a clear linear trend in the data in keeping with other RT studies^22^. The linear regression analysis confirms this impression: a single regression line with a slope below unity (b = 0.75) quite well explains the data of both children with dyslexia and controls (R^2^ = 0.98). Thus, data from children with dyslexia do not deviate from the distribution of controls but are distributed on the same state trace, although they cluster to the upper right of the plot.

An under-additive tendency is present in the data such that the group differences increase with decreasing reading times. Thus, the advantage for stimuli in multiple displays is greater for typically developing readers (with faster reading times) than for children with dyslexia (with slower reading times closer to the diagonal equality line) as well as for easier (such as reading 5-letter words) than more difficult conditions (such as 7-letter pseudowords). Conversely, slower reading times are generally associated with smaller differences between single and multiple display conditions. Notably, in no case an inversion of this effect is present.

## General discussion

Firstly, results confirmed that both control readers and children with dyslexia were able to process stimuli in a faster way in the case of multiple displays^16,17^. Therefore, both groups can partially overlap the various processes involved in dealing with multiple targets (as typical of standard reading). Using the DEM^19^ as a general reference, the faster responses in the case of multiple displays were associated with a reduction in the weight of the sensory-motor component on the response time but not with a reduction of inter-individual variability. Data fell on separate regression lines, indicating that a single factor cannot account for performance in single and multiple displays. As shown in Fig. 1, this overall shift may indicate the capacity to overlap the non-decisional component of the response (sensory-motor compartment in the DEM terminology^19^). This finding confirms previous observations based on the single-word presentation that children with dyslexia are selectively impaired in the decisional part of the response and not in the sensory-motor one^24,30^.

Secondly, there are several indications in the present data that the requirement to read sequentially taxes performance by augmenting the relative impact of the experimental manipulations used. The spread among the means for the various experimental manipulations (i.e., short and long words, words and pseudowords) is larger in the case of multiple display conditions than in the case of single display conditions, indicating that the increase is of a multiplicative nature. Thus, the effect of length is stronger when reading sequentially than when reading isolated words. Notably, this effect is not evident in the raw data as performance in the multiple display conditions is generally faster, but becomes apparent when we examine data with the means/SDs (Fig. 5) or Brinley (Fig. 6) plots. The taxing effect of sequential reading is in keeping with the proposal of cascaded processing advanced by Protopapas et al.^15^.

Thirdly, a single global factor accounts well for the group differences across the multiple-display conditions. Therefore, the taxing component due to the requirement to read sequentially is present for both groups of children, although proportionally more evident in the case of children with dyslexia. This latter finding is apparent in the case of the State trace plot (Fig. 7). The reading advantage from multiple-display conditions is larger for easier conditions (such as short words) and faster, control subjects and smaller for more difficult conditions (such as long pseudowords) and slower, dyslexic subjects. In other terms, it shows an under-additivity tendency. This global tendency contrasts with the over-additive effect observed in the comparison between groups (as in the Brinley plot; Fig. 6), such that group differences are larger for slower reading times, either because of more difficult conditions or slower individual performances. These two tendencies tend to cancel out each other in the raw data, generating the inconsistent tendencies noted in the raw data presented in Fig. 4.

Fourthly, one interesting finding in the case of multiple displays is that, in the Brinley plot, the global factor has an intercept very near zero (see Fig. 6). In reading single isolated words, the global factor is identified by linear regression with a slope, indicating the degree of the slowness of the children with dyslexia, and an intercept expressing, according to the DEM^19^, the part of the response connected to the sensory-motor compartment. Thus, one may hypothesize that the absence of an intercept in the multiple display condition is not just an accidental finding but indicates that the sensory-motor component is absorbed in the overlap of different processes. Consequently, it no longer plays a direct influence on the response pattern. If correct, this interpretation would have interesting implications also for the clinical evaluation of reading. In a previous paper, we proposed that using a ratio may prove particularly useful in capturing the multiplicative nature of the differences between the performances of children with and without a reading deficit^21^. By contrast, measures of effect size based on standard parametric analyses (such as Cohen’s d) fail to capture this fundamental characteristic of the reading deficit. The same applies to the cut-offs (frequently used at an individual level) based on standardized scores (such as a -2.0 z-score below the normative sample). These analyses erroneously assume homogeneity of variance across tests and groups^21^. On the contrary, inter-individual variability closely covaries with mean performance, indicating multiplicative differences between children with dyslexia and control readers. We propose that clinical measures of the reading disorder should consider this fundamental characteristic of the reading fluency deficit. This may entail developing norms for reading time measures based on ratio values that a) capture the multiplicative nature of the disturbance and b) may be easily applied to standard tasks of reading texts.

Overall, the present findings indicate that the requirement to read sequentially poses a stress on reading performance, potentiating the impact of experimental manipulations, such as lexicality and stimulus length. This effect is present for control readers and children with dyslexia but is proportionally more pronounced in these latter children as general processing differences are fully nested with performance differences due to experimental manipulations.

There are various reasons to believe that, for a minority of children, standard text reading represents the “perfect storm”. First, in reading, individual differences in performance are very tightly associated with general stimulus difficulty, indeed more than with any other type of materials/tasks^31^. Accordingly, even moderate increases in stimulus difficulty bring about proportionally large increases in inter-individual variability, i.e., particularly large effects on the tail of the distribution, which is critical in the case of children with dyslexia^31^. Further, the difficulty in decoding words is amplified by the need to carry out the overall task with an inherent time pressure (due to the requirement to synchronize processing with the timing of saccadic exploration) as well as the need to coordinate several sub-componential tasks (such as holding the word in memory for later pronunciation^32^). Evidence indicates that most children with dyslexia are unimpaired in the various sub-components of reading per se. For example, no selective deficits are present in programming saccades^33–35^ or in carrying out corrective saccades^36^. However, as shown here, the need to carry out these multiple sub-tasks together boosts the impact of stimulus complexity (compared to single-word reading), amplifying its impact on performance, particularly in children with dyslexia. In sum, text reading represents a compound of complexities for children with dyslexia: on the one hand, stimulus decoding is particularly complex in the case of sequential reading and on the other, the need to synchronize decoding with eye fixation poses a tight timing constraint to this processing.

Up to date, studies on reading and dyslexia focussed on letter/word level with little interest in integrating this information with the processing of multiple stimuli characteristic of standard reading as well as clinical reading testing. Very recently, work on the relationship between different levels of processing in reading is starting to come out ^37,38^. In this vein, the present findings help shorten the gap between experimental measures of reading performance typically based on a reading single, isolated words, and standard, as well as clinical measures of reading, which require the processing of multiple stimuli.

## Conclusions

This study helps to understand reading from multiple displays, a condition more like real-life situations than the single display presentation used in most experimental studies. The results indicated an advantage in reading times for multiple over single displays. Based on different models of global processing, we propose that this advantage may be ascribed to the capacity to overlap the non-decisional component of the response, an effect present both in children with dyslexia and in control readers. Importantly, the spread among the means for the various experimental manipulations was larger for the multiple than the single display conditions, amplifying dyslexics’ impairment over difficult conditions. Therefore, the requirement to read sequentially appears to potentiate the relative impact of the experimental manipulations (i.e., lexicality and length). This taxing effect of sequential reading was present in both groups of children but proportionally more evident in the case of children with dyslexia.

## Materials and methods

### Participants

Participants included 32 typically developing readers and 23 children with dyslexia attending 6th grade and with performance within normal limits on the Coloured Progressive Matrices of Raven (CPM^39^). Groups were comparable for gender (χ^2^ < 1), age, and Raven CPM score (Fs < 1). The children with dyslexia scored at least 1.65 SD below the norm for either reading time or accuracy on a standardized reading test (MT Reading test^40,41^). The research was reviewed and approved by the Ethics Committee of Santa Lucia Foundation, Rome, Italy. Written informed consent to participate in this study was provided by the participant’s legal guardian/next of kin. All methods were performed by the ethical standards of the Declaration of Helsinki.

### General design

To investigate single-multiple word display relationships, we set out the goal of overcoming some methodological criticisms. Firstly, we created experiments in which single and multiple conditions would be directly comparable. In the multiple condition, an array of 25 words was simultaneously displayed while, in the single condition, 25 words appeared one at a time from left to right, line by line (in a five columns x five rows arrangement). We decided to include sound and visual markers for the single display condition to eliminate any possible confounding due to the location and appearance of the target stimulus. Moreover, since in the single display conditions, the visual attention is captured in a bottom-up fashion by a salient external trigger^17^, we wanted to emphasize this facilitation. Notably, this condition differs from the multiple one in which the subject is not driven by an external trigger but must find an internal pace of its own to read the stimuli fluently. To capture a measure of a proper mental process, we excluded from the analysis the trials in which the observer made a reading error.

### Assessment measures

*Non-verbal IQ assessment*. Non-verbal IQ level was assessed using the Raven’s Coloured Progressive Matrices. All children scored well within the normal limits (> 5th percentile) according to the Italian norms^39^.

*Reading Assessment*. The reading test used for screening purposes was the MT Reading Test^40,41^: the child is given a passage and is requested to read it as fast and accurately as possible. The child reads a passage aloud within a 4-min time limit. Reading time (s/syllable) and accuracy (number of errors, adjusted for text read) are scored^40,41^. Data are presented in Table 1 both as absolute and normalized values.

**Table 1:** Summary statistics for the two groups of participants: N, number of female and male participants; mean age (in years) and SDs; Raven’s Coloured Matrices test: mean raw scores, SDs, and mean percentiles; MT Reading test: mean raw scores and SDs on reading time (s/syll.) and accuracy measure (errors over 10 multiple choice questions); Words/Non-words Reading Test: mean raw scores (s to read a 30 item list), SDs, and mean z scores separately for six separate reading conditions; One Minute reading Test: Mean Words per minute and SDs.

	Children with dyslexia				Control children			
N	23				32			
Male	13				17			
Female	10				15			
	M		SD		M		SD	
Age	11.6		0.4		11.4		0.3	
Raven test	29		4		32		3.4	
Mean percentile	56.6				75.8			
MT Reading test								
Reading time (s/syll.)	0.41		0.15		0.21		0.02	
Accuracy (errors)	22		7.8		6.2		2.8	
Words/non-words reading test								
Short high-frequency words	24.1		9		13.8		2.1	
Mean Z score*	− 0.6				1.09			
Long high-frequency words	35.9		15.8		17.5		3.0	
Mean Z score	− 1.5				1.3			
Short low-frequency words	31.7		11.9		15.9		2.9	
Mean Z score	− 1.4				1.1			
Long low-frequency words	61.4		22.7		25.1		4.9	
Mean Z score	− 2.5				1.1			
Short on-words	39.2		15.3		18.1		3.3	
Mean Z score	− 1.5				1.1			
Long non-words	68.2		23.2		35.4		8.5	
Mean Z score	− 1.5				1.0			
One minute reading test Words per minute	56.5		16.8		102.4		12.7	

*A - sign indicates lower performance (i.e., slower reading times)

Two tests requiring the reading of lists of words were used. One was the One Minute test (OMrT-adaptation of the One Minute Reading test^42^). We extracted 158 Italian low-frequency words from the Children Word frequency database^43^. The words were presented simultaneously in a grid format, like the RAN tests. Words were all bi-syllabic and 5-letter long, with a frequency of occurrence lower than 30 over 1 million (mean = 15.54, SD = 6.44; range 6–30). Children had to read aloud the words as quickly as possible, from left to right, across the page in one minute. The score is the total number of words read correctly (Table 1).

The other test was the *Words and Non-words Reading Test*^44^. This test features six lists of 30 stimuli varying in frequency, lexicality, and length; norms available for each of the six sub-lists were used (Table 1).

*Experimental task*. Stimuli were selected from the Varless2 database^45,46^. For both serial and discrete tasks, the conditions were composed of six blocks of stimuli. Three blocks contained 25 words each; in one block, there were 5-letter words, in one, 7-letter words, and, in one, both 5- and 7-letter words. The three blocks of pseudowords were similar in terms of the number of stimuli and characteristics of stimulus length. Pseudowords were derived by the words, substituting 2–3 letters. They maintained the same syllabic structure and the first phoneme of derived words. The word subsets had a general mean frequency of 54.87 (SD = 75) according to the children’s word frequency count^43^ and did not differ from each other for the presence of consonant clusters, geminate consonants, contextual rules, letter confusability, stress, point of articulation of the first phoneme, age of acquisition, imageability, children’s word frequency and bigram frequency (according to^46^). Furthermore, the two parallel lists used for the single- and multiple-display conditions did not differ for any psycholinguistic variable.

There were four experimental blocks in each condition (single and multiple displays); each involved reading either words or pseudowords (of variable length).

- *Multiple-display conditions:* an array of 25 words was statically displayed on a laptop screen. Children had to read aloud all words as quickly as possible, working from left to right and from top to bottom.
- *Single-display condition:* 25 words (or pseudowords) appeared one at a time on the screen, from left to right, marked by a sound (“beep”). Children had to read aloud the target stimuli as quickly as possible. Every word/pseudoword appeared after 3 s from the onset of the previous stimulus. Once the words appeared, they remained on the screen until the child’s response (or for a maximum of 3 s).

Words and pseudowords appeared in black lowercase Courier font (characterized by a constant centre-to-centre letter spacing) on a white background.

Participants were tested individually in a quiet room at their school. They were seated 57 cm away from the screen, and the x-width measured 1.06 deg. For both multiple and single displays, item presentation and response recording were controlled by the MATLAB & Simulink R2015b experimental software.

### Data scoring and analysis

*Scoring*. In each condition, we counted the number of reading errors. Utterances that did not correspond to the presented word, self-corrections, stress errors, and word omissions were considered errors. We determined offline the total reading times for multiple and single display conditions using the audio editor and recorder Audacity 2.1.3 Version. The dependent variable was the total reading time of correct responses: total reading times (RTs + pronunciation times) for the single-display condition and PAUSE + pronunciation times in the multiple display conditions.

For the single-display condition, the total reading time of all items was considered, including both onset latency and articulation time. The pauses between the end of the pronunciation and the next “beep” as well as the errors, were eliminated. In the event in which the child could not read the word (or pseudoword) within 3 s before the next word appeared, the reading times of the unread word (or pseudoword) and those of the next word (or pseudoword) were deleted. Sometimes, the reading of two stimuli overlapped in the same window of time (that is, the child read the word, or pseudoword, after the next appearance) to create a situation of multiple reading. In these cases, we eliminated both times. The total reading time of each condition (5-letter words, 5- and 7-letter mixed words, and 7-letter words) was divided by the number of words read correctly to obtain an estimate of “time per item”.

For the multiple display conditions, the total reading time of all items was considered, including pauses and articulation time. We eliminated errors from the total reading time calculation. We divided the total reading time of each condition by the number of words read correctly to obtain an estimate of “time per item”.

*Detection of global components*. Differences between typically developing readers and dyslexic children have been explored from a global (i.e., a non-task-specific) perspective.

First, we tested the DEM’s^19^ prediction of a linear relationship between the overall group means in total reading times and the SDs over the same conditions. As mentioned above, according to the DEM, this type of plot is critical to detect a global factor. Note that DEM aims to account for RTs, not articulation times. Previous evidence^25^ indicates that articulation time does not merely add a constant, because it varies with the stimulus condition (i.e., length of words). Therefore, we expected a distribution different from that originally foreseen by Myerson et al.^19^.

Second, to detect global components in group differences, we tested the prediction of a linear relationship between the condition means of the group of children with dyslexia versus the group of control children using a *Brinley plot*. This relationship is diagnostic of the over-additivity effect. The slope of the linear relationship represents an estimate of the severity of the impairment.

Finally, we used the *State trace* analysis^22,29^ to examine whether the reading performance of children with dyslexia and control readers in single and multiple displays can be ascribed to a single latent dimension. The means of the multiple display conditions are plotted against those of the single display conditions separately for typically developing readers and children with dyslexia. Thus, variation in the plot is related to the causal variables (lexicality and lengths) in combination with the grouping variable. The presence of a single monotonic response function would be in keeping with the idea that a single latent dimension accounts well for the functioning of the system. As stated above, the state trace does not assume linearity in the relationship between causal and indicator variables. Yet, the application of this method to RTs has often produced linear state traces^23^. Accordingly, the resulting plot will be inspected for linearity, and a linear regression model will be tested if appropriate.

In each case, we aimed to establish how, if present, the global components in the data would vary as a function of the experimental manipulation (single, multiple displays) and of the group (children with dyslexia vs. control readers).

### Ethical statement

All methods were performed by the ethical standards of the Declaration of Helsinki.

### Commercial relationships

Conforti S., Marinelli C.V., Zoccolotti P., and Martelli M., None.

### Supplementary Information

Supplementary Information.

## Data Availability

The datasets generated during and/or analysed during the current study are available from the corresponding author.
