# Supplementary material for: The metrics of reading speed: understanding developmental dyslexia
Source: Sci Rep. 2024 Feb 19;14:4109. doi: 10.1038/s41598-024-52330-x (PMC10876942; doi:10.1038/s41598-024-52330-x)
Supplement: Supplementary file 1 — Supplementary Information. [file 41598_2024_52330_MOESM1_ESM.docx]

**The metrics of reading speed: understanding developmental dyslexia**

**Sara Conforti^1^, Chiara Valeria Marinelli^2*^, Pierluigi Zoccolotti^1,3^, and Marialuisa Martelli^1,3^**

**Supplementary materials**

**Model of human information processing focussing on global processing**

The “*difference engine model*” (DEM)

The DEM^1^ aims to account for individual variability in RTs to timed tasks assuming two different and independent portions of the response, the “cognitive-decision” and “sensory-motor” compartments.

The model revolves around the plot that binds the means of the experimental conditions and their corresponding standard deviations. The link between them is considered as a rule or law underlying individual performance in speeded tasks. According to the DEM, near-linearity is expected between condition means with their corresponding standard deviations. The slope of this linear relationship (beta) would indicate the correlation between the cognitive stages hypothetically involved in the task (intended as a series of generic computational steps). This part of the response is the one marking the “cognitive” compartment of the reaction time measures. In keeping with the idea that the cognitive compartment is described by a single general “rule”, the DEM predicts that the performance of groups with different overall processing speeds would lie on the same regression line (although in different parts of the curve).

The sensory-motor compartment can be estimated based on the x-axis in the plots contrasting condition means with their corresponding standard deviations. This portion of the response is unrelated to the cognitive compartment (hence, unaffected by manipulations affecting task difficulty along cognitive dimensions) and not different in groups varying for a global speed factor (such as young adults and elderly). Consistently with these predictions, much evidence indicates that this pattern holds when older and younger adults are compared on a large variety of timed tasks^2^. However, the DEM does not consider the execution component itself in timed tasks (i.e., the time to utter the word in the case of reading). For this reason, in the present study, the predictions of this model represent a general framework but cannot be directly used to account for variability in total reading times.

*The rate and amount model (RAM)*

The RAM proposes that the general cognitive speed of the individual (rate) and the difficulty of a given condition (amount)^3^ interact multiplicatively to determine individual RTs and produce larger group differences in more difficult conditions (over-additivity effect). This can be shown in the so-called Brinley plot, in which the RT means of a slower group (e.g., older adults) across experimental conditions are plotted as a function of the RT means of the faster group (younger adults) over the same conditions. Results indicate that group differences in timed tasks can be ascribed to one (or few) global factor(s)^3,4^.

*The State trace analysis*

The State trace analysis aims to compare two critical conditions across populations yielding different overall levels of performance and inter-individual variability.

Bamber^5^ originally described this analysis as a relatively simple graphic way to define the space for a given process: the State trace plot allows an understanding of whether a set of variables can be ascribed to a single latent variable or to more variables ^5,6^.

While much research has focused on examining the same dependent variable under various experimental conditions, the influence of a grouping variable over the latent variable has also been examined with this approach. One study on reading^7^ used State trace analysis to investigate the putatively specific deficit in reading pseudowords in children with dyslexia^8-10^. Grouping (i.e., reading disabled versus typical children) was among the causal variables along with lexicality and item difficulty; the indicator variables were the reading times for words and pseudowords. It was hypothesized that, if the same reading process is responsible for both groups as a function of the causal variables, all data points should be on one monotonically increasing response function^7^. Alternatively, if the reading groups’ underlying reading mechanisms differ, each group will have its own response function. Results were consistent with the former prediction, indicating no specific deficit for reading pseudowords in Dutch disabled readers^7^.

References

1. Myerson, J., Hale, S., Zheng, Y., Jenkins, L., & Widaman, K. F. The difference engine: A model of diversity in speeded cognition. *Psychon. Bull. Rev.* **10**, 262-288 (2003).
2. Zheng, Y., Myerson, J., & Hale, S. Age and individual differences in visuospatial processing speed: Testing the magnification hypothesis. *Psychon. Bull. Rev.* **7**, 113-120 (2000).
3. Faust, M. E., Balota, D. A., Spieler, D. H., & Ferraro, F. R. Individual differences in information-processing rate and amount: implications for group differences in response latency. *Psychol. Bull.* **125**, 777-799 (1999).
4. Verhaeghen, P., & Cerella, J. Aging, executive control, and attention: A review of meta-analyses. *Neurosci. Biobehav. Rev.* **26**, 849-857 (2002).
5. Bamber, D. State-trace analysis: A method of testing simple theories of causation. *J. Math. Psychol.* **19***,* 137-181 (1979).
6. Prince, M., Brown, S., & Heathcote, A. The design and analysis of state-trace experiments. Psychol. Meth. **17**, 78-99 (2012). <https://doi.org/10.1037/a0025809>
7. Van den Broeck, W., & Geudens, A. Old and new ways to study characteristics of reading disability: The case of the nonword-reading deficit. *Cogn. Psychol.* **65**, 414-456 (2012).
8. Herrmann, J. A., Matyas, T., & Pratt, C. Meta-analysis of the nonword-reading deficit in specific reading disorder. *Dyslexia,* **12***,* 195–221 (2006). <http://dx.doi.org/10.1002/dys.324>
9. Rack, J. P., Snowling, M. J., & Olson, R. K. The nonword reading deficit in developmental dyslexia: A review. *Read. Res. Quart.* **27**, 29-53 (1992).
10. Van Ijzendoorn, M. H., & Bus, A. G. Meta-analytic confirmation of the nonword reading deficit in developmental dyslexia. *Read. Res. Quart.* **30***,* 267-275 (1994). <http://dx.doi.org/10.2307/747877>.

|  |  |  | Single-display | | Multiple-display | |
| --- | --- | --- | --- | --- | --- | --- |
|  |  |  | Mean | SD | Mean | SD |
| Control children | Words | 5-letter | 1205 | 151 | 527 | 85 |
|  |  | 5/7-letter | 1311 | 155 | 541 | 76 |
|  |  | 7-letter | 1346 | 154 | 628 | 121 |
|  | Pseudowords | 5-letter | 1318 | 149 | 748 | 159 |
|  |  | 5/7-letter | 1499 | 178 | 882 | 198 |
|  |  | 7-letter | 1561 | 181 | 1076 | 260 |
| Children with dyslexia | Words | 5-letter | 1572 | 357 | 956 | 391 |
|  |  | 5/7-letter | 1809 | 440 | 1143 | 493 |
|  |  | 7-letter | 1904 | 478 | 1377 | 590 |
|  | Pseudowords | 5-letter | 1962 | 558 | 1528 | 675 |
|  |  | 5/7-letter | 2249 | 618 | 1792 | 805 |
|  |  | 7-letter | 2434 | 636 | 2180 | 928 |

**Table S1**. Mean (and SDs) reading times (in ms) as a function of stimulus length, lexicality, type of display, and group.
